# Klinefelter syndrome beyond hypogonadism: multisystem manifestations and a framework for clinical surveillance

**DOI:** 10.3389/fendo.2026.1848181

**Published:** 2026-06-24

**Authors:** İnan Anaforoğlu

**Affiliations:** Department of Endocrinology and Metabolism, School of Medicine, Acıbadem Mehmet Ali Aydınlar University, Istanbul, Türkiye

**Keywords:** 47,XXY, bone mineral density, epigenetics, INSL3, Klinefelter syndrome, metabolic syndrome, mortality, neurocognition

## Abstract

Klinefelter syndrome (KS) is the most common male sex chromosome aneuploidy, affecting approximately 1 in 450–600 male births. Characterised by a 47,XXY karyotype in the majority of cases, it is widely known for its association with hypergonadotropic hypogonadism, infertility, and impaired testicular function; however, the clinical consequences of the supernumerary X chromosome extend far beyond the reproductive axis. This review provides an updated and integrative overview of the multisystem involvement in KS, with particular emphasis on metabolic derangements, cardiovascular and thromboembolic risk, skeletal health, malignancy patterns, thyroid dysfunction, and neurocognitive outcomes. Emerging concepts, including the bone-testicular axis, the role of insulin-like peptide 3 (INSL3), and osteocalcin-mediated endocrine crosstalk, are critically appraised. Recent data on mortality, accelerated biological ageing, and the systemic impact of testosterone replacement therapy (TRT) are discussed in light of contemporary evidence. Given the substantial burden of comorbidities and the high rate of underdiagnosis, early recognition and implementation of a structured, multidisciplinary surveillance strategy are essential to improve long-term outcomes in individuals with KS.

## 1 Introduction

Klinefelter syndrome (KS) was first described by Harry Klinefelter in 1942 and represents the most common chromosomal sex aneuploidy in males. The classic karyotype is 47,XXY, present in approximately 90% of cases, with the remaining 10% carrying mosaic forms (46,XY/47,XXY) or higher-grade aneuploidies such as 48,XXXY or 49,XXXXY. The estimated prevalence is 1 in 450–600 male births; however, population data from Denmark indicate that up to 75% of affected individuals remain undiagnosed throughout their lifetime, with the mean age at diagnosis approximating 25 years (1, 2).

The additional X chromosome disrupts genetic and cellular homeostasis, most prominently causing testicular dysgenesis through hyaline degeneration of the gonads, resulting in small, firm testes. This process accelerates at puberty, leading to primary hypogonadism with elevated FSH, LH, and SHBG, reduced total and free testosterone, elevated estradiol, and markedly decreased inhibin and AMH. Severity correlates with the number of supernumerary X chromosomes (3).

Importantly, phenotypic variability among individuals with KS cannot be fully explained by androgen deficiency alone. Mosaic forms (46,XY/47,XXY) often exhibit milder phenotypes, including higher testosterone levels and better preserved spermatogenesis. In contrast, higher-grade aneuploidies are associated with more severe neurodevelopmental impairment and systemic involvement. These observations suggest that gene-dosage effects interact with epigenetic regulation and X-inactivation patterns in a non-linear fashion (4, 5).

Approximately 15% of genes on the extra X chromosome escape X-inactivation, leading to their overexpression. These genes may influence immune regulation, metabolism, and neurodevelopment. Emerging evidence also suggests that DNA methylation patterns and epigenetic ageing markers contribute to disease heterogeneity, although their precise role remains to be elucidated (5, 6).

This review synthesises current evidence regarding the non-reproductive consequences of KS, discusses their underlying mechanisms, which involve both the direct gene-dosage effect of the extra X chromosome and downstream hormonal perturbations, and outlines evidence-based surveillance and treatment strategies.

### 1.1 Literature search strategy

This narrative review was conducted through a systematic search of PubMed/MEDLINE. Search terms included “Klinefelter syndrome”, “47,XXY”, and “sex chromosome aneuploidy”, combined with domain-specific terms covering metabolic, cardiovascular, skeletal, thyroid, neurocognitive, oncological, and psychosocial manifestations. Articles were selected on the basis of clinical relevance, methodological quality, and recency, with preference given to prospective cohort studies, randomised controlled trials, systematic reviews, and meta-analyses. The European academy of andrology (EAA) 2021 guidelines.

**Figure 1:**
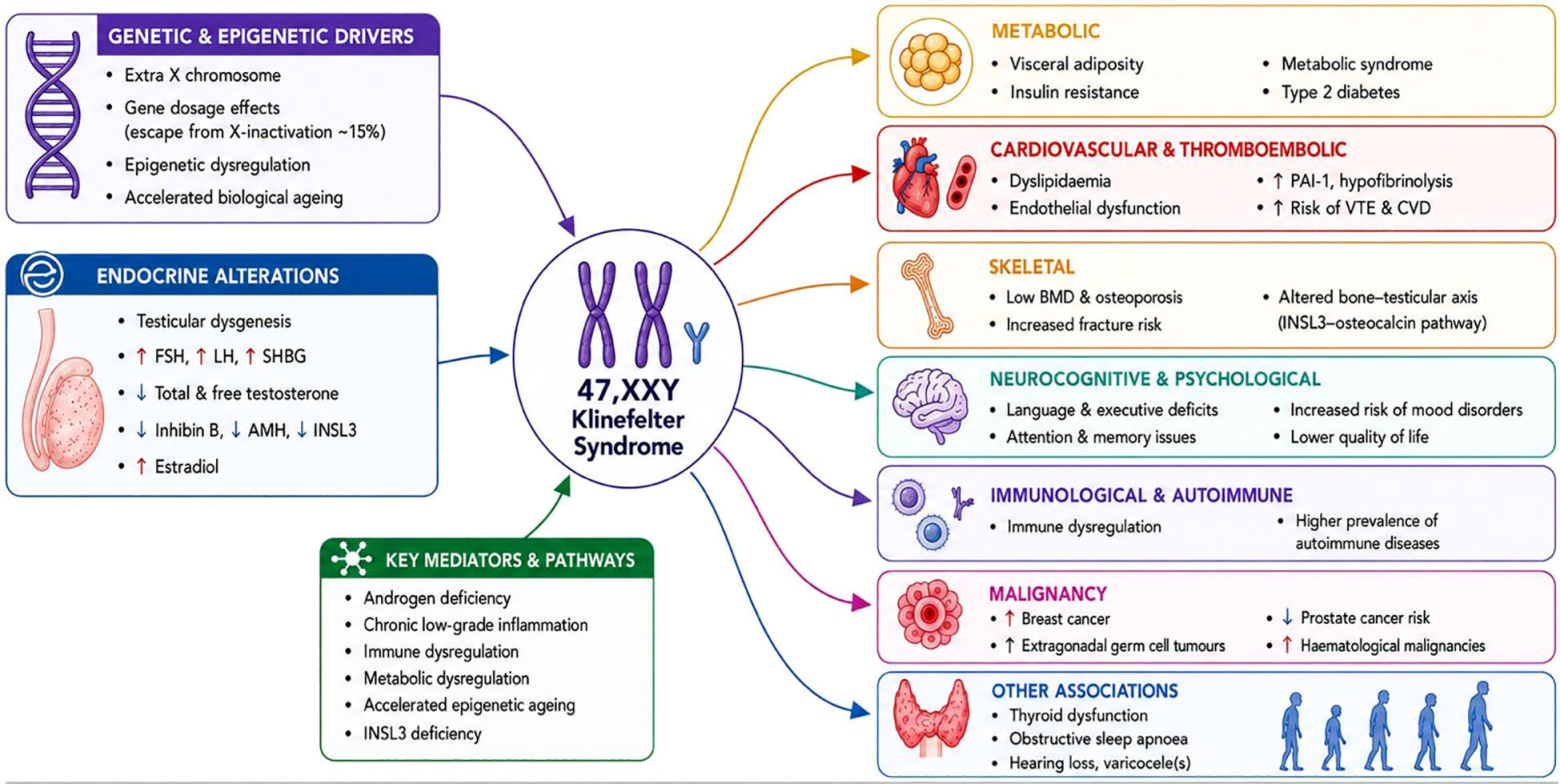
Klinefelter syndrome (47,XXY): multisystem manifestations and pathophysiological links. Klinefelter syndrome (KS) arises from a supernumerary X chromosome (47,XXY) and behaves as a multisystem disorder rather than an isolated reproductive condition. At the molecular level, gene dosage effects, driven by genes that escape X-chromosome inactivation together with epigenetic dysregulation and accelerated biological ageing constitute the upstream drivers of the phenotype. These converge on a characteristic endocrine signature of primary (hypergonadotropic) hypogonadism: testicular dysgenesis with elevated FSH, LH and SHBG, reduced total and free testosterone, low inhibin B, AMH and INSL3, and a relative increase in estradiol. Androgen deficiency, chronic low-grade inflammation, immune and metabolic dysregulation, accelerated epigenetic ageing and INSL3 deficiency act as the key mediators linking this genetic and endocrine background to downstream organ involvement. Through these pathways, KS predisposes to a broad spectrum of clinical consequences: metabolic disturbance (visceral adiposity, insulin resistance, metabolic syndrome and type 2 diabetes); cardiovascular and thromboembolic risk (dyslipidaemia, endothelial dysfunction, elevated PAI-1 with hypofibrinolysis, and an increased risk of venous thromboembolism [VTE] and cardiovascular disease [CVD]); skeletal compromise (low bone mineral density, osteoporosis and increased fracture risk, reflecting an altered bone-testicular axis via the INSL3-osteocalcin pathway); neurocognitive and psychological features (language and executive deficits, attention and memory difficulties, an increased risk of mood disorders and lower quality of life); immunological and autoimmune dysregulation, with a higher prevalence of autoimmune diseases (e.g., systemic lupus erythematosus, rheumatoid arthritis and type 1 diabetes mellitus); an altered malignancy profile (increased risk of breast cancer, extragonadal germ cell tumours and haematological malignancies, alongside a reduced prostate cancer risk); and other associations including thyroid dysfunction, obstructive sleep apnoea, hearing loss and varicocele(s). *AMH*, *anti-Müllerian hormone*; *BMD, bone mineral density*; *CVD, cardiovascular disease*; *FSH, follicle-stimulating hormone*; *INSL3*, *insulin-like peptide 3*; *LH*, *luteinising hormone*; *PAI-1*, *plasminogen activator inhibitor-1*; *RA, rheumatoid arthritis*; *SHBG*, *sex hormone-binding globulin*; *SLE*, *systemic lupus erythematosus*; *T1DM*, *type 1 diabetes mellitus*; *VTE*, *venous thromboembolism*.

To illustrate the multisystem nature of KS, we developed a schematic representation (**Figure 1**) summarizing the interplay between genetic, endocrine, metabolic, and immunological factors.

The figure emphasizes that Klinefelter syndrome extends beyond reproductive dysfunction, encompassing neurocognitive, autoimmune, and thromboembolic domains. This integrated perspective supports a multidisciplinary approach to management and highlights the need for early diagnosis and long-term follow-up.

## 2 Metabolic syndrome, body composition, and diabetes risk

Men with KS carry a substantially elevated risk of metabolic syndrome, with a reported prevalence of 34-44% compared with approximately 10% in healthy male controls. Importantly, metabolic abnormalities have also been identified in prepubertal boys with KS (prevalence approximately 24%), indicating that the chromosomal aneuploidy itself, beyond hypogonadism, contributes to this phenotype (7, 8).

Bojesen et al. demonstrated in a landmark study that 44% of KS men had metabolic syndrome, associated with significantly lower androgen concentrations, reduced HDL cholesterol and insulin sensitivity, and higher total fat mass, LDL cholesterol, triglycerides, and C-reactive protein. The same study showed that testosterone replacement therapy (TRT) reduced LDL cholesterol and altered adiponectin levels but did not produce significant improvements in body composition in the short term. Truncal adiposity, driven by hypogonadism, was identified as the principal determinant of insulin sensitivity and metabolic syndrome in KS (8).

The pathophysiology is multifactorial. The additional X chromosome directly impairs androgen signalling pathways, while the resultant hypogonadism reduces lipolysis, increases visceral adipose tissue accumulation, and decreases muscle mass and tone. Consequently, men with KS characteristically exhibit truncal obesity, increased total body fat, and reduced lean muscle mass, even when body weight appears normal. These abnormalities have been identified even in prepubertal individuals, suggesting that chromosomal factors contribute independently of overt hypogonadism (7, 9). Early investigations in prepubertal KS boys (aged 4-9.5 years) have explored the relationship between Sertoli cell function -as reflected by anti-Müllerian hormone (AMH) and inhibin B- and metabolic syndrome features. Reduced Sertoli cell activity, evident even before the pubertal gonadal decline, correlates with a higher number of metabolic syndrome components, suggesting that gonadal insufficiency begins early and contributes to metabolic dysregulation long before clinical hypogonadism becomes apparent (7, 10).

Gravholt et al. reviewed evidence on body composition, metabolic syndrome, and type 2 diabetes mellitus (T2DM) in KS, summarizing data from prior cohort studies. The study demonstrated a markedly higher prevalence of T2DM, impaired fasting glucose (IFG), and metabolic syndrome in KS subjects relative to controls, with metabolic syndrome affecting approximately 46% of KS patients. The authors concluded that abdominal obesity and hypogonadism are key contributors to the increased cardiometabolic risk observed in men with KS, highlighting the importance of routine metabolic screening in this population (11). A British cohort mortality study of 3,518 men with KS identified a standardised mortality ratio (SMR) for diabetes of 5.8 (95% CI: 3.4-9.3), underscoring the lethal potential of this comorbidity (12).

These sentences should be amended/re-arranged as: A longitudinal study evaluating diabetes prevalence in KS reported diabetes in 12.5% of patients with 47,XXY karyotype, rising to 57.1% in those with atypical karyotypes such as 46,XY/47,XXY mosaicism (13). T2DM risk is markedly elevated in KS and has been consistently documented in the medical literature. A study examining comorbidities in type 1 diabetes mellitus (T1DM) patients found that KS was present in 1.9% of T1DM cases (14). Autoimmune markers including IA-2 antibodies and anti-GAD antibodies were found in approximately 9.8% of men with KS compared with 6.5% of healthy male controls, suggesting susceptibility to immune-mediated pancreatic beta-cell injury (15). It may be critical to seek T1DM especially in the pediatric population.

Current guidelines EAA recommend that all patients with KS undergo regular assessment of body composition, fasting plasma glucose, HbA1c, and lipid profile from early adulthood. Lifestyle modification, including dietary counselling and structured physical activity, is the cornerstone of metabolic risk reduction. TRT improves insulin resistance in obese patients with T2DM and may reduce waist circumference and improve lipid profiles in older men, though its effect on body composition is inconsistent. Pharmacological management of established T2DM, hypertension, and dyslipidaemia should follow standard evidence-based guidelines (16).

## 3 Cardiovascular disease and thromboembolic risk

### 3.1 Vascular endothelial dysfunction

Patients with KS demonstrate a significantly elevated cardiometabolic risk that extends beyond classical hypogonadism. Evidence suggests that this risk originates early in life, potentially as far back as the intrauterine period, and is shaped by multiple interacting factors including androgen receptor (AR) CAG repeat length, reduced fetal testosterone exposure, and postnatal hypogonadism (17, 18). Even in young KS patients without established metabolic or cardiovascular disease, endothelial dysfunction and insulin resistance are already measurable, with hypogonadism identified as a key contributing factor (19). More recently, Demirci et al. demonstrated that the triglyceride-glucose (TyG) index -a simple surrogate marker of insulin resistance- is significantly elevated in KS patients compared to healthy controls, and is independently associated with endothelial dysfunction as reflected by elevated asymmetric dimethylarginine levels, suggesting its utility as a practical screening tool in this population (20). Di Mambro et al. were the first to investigate circulating endothelial progenitor cells (EPC) which are bone marrow-derived cells that maintain vascular integrity and serve as independent predictor of cardiovascular morbidity and mortality in KS. Studying 68 adult men with 47,XXY KS and 46 healthy controls, the authors found that KS patients without conventional cardiovascular risk factors had EPC levels comparable to KS patients with risk factors, and significantly lower than healthy controls without risk factors. Androgen replacement therapy over six months did not restore EPC counts in hypogonadal patients. These findings suggest that the intrinsic vascular vulnerability in KS may be partially independent of testosterone deficiency alone. The supernumerary X chromosome itself may contribute to impaired endothelial repair capacity and elevated cardiovascular risk, independent of the hormonal milieu (21).

The cardiometabolic burden in KS is further compounded by unfavorable changes in body composition, including increased visceral adiposity and ectopic fat deposition. Granato et al. investigated epicardial fat accumulation in KS for the first time, demonstrating that hypogonadal KS men exhibit epicardial fat deposition comparable to obese age-matched euploid subjects. Notably, the supernumerary X chromosome alongside BMI emerged as one of the strongest independent determinants of both epicardial and truncal fat, with testosterone levels and TRT showing no significant effect. This finding reinforces the concept that structural cardiovascular risk in KS extends beyond hypogonadism (21, 22). Collectively, these data indicate that cardiovascular and metabolic risk assessment should be initiated early in KS patients, regardless of age or current symptom burden, and that hypogonadism-targeted interventions may offer a meaningful opportunity to attenuate this risk.

### 3.2 Congenital cardiac anomalies

The association between KS and cardiovascular disease has been recognized for decades. Rosenthal et al. was among the first to formally document a link between KS and congenital cardiac malformations, while Fricke et al. subsequently demonstrated through echocardiographic evaluation that mitral valve prolapse was present in 55% of KS patients, nearly nine times the rate observed in the general male population, carrying implications for arrhythmic risk and infective endocarditis (23, 24). More recently, Salem et al. characterized the clinical profile of men with acquired long QT syndrome and torsades de pointes associated with hypogonadism, demonstrating that testosterone deficiency, regardless of its aetiology, may constitute a reversible risk factor for potentially fatal ventricular arrhythmias (25). While this association has not been studied specifically in KS, it carries relevant implications for affected men given their near-universal hypogonadism. Notably, however, KS-specific electrocardiographic data suggest a more nuanced picture: Jørgensen et al. demonstrated that KS men exhibit shorter QTc intervals compared with healthy controls, with TRT further shortening the interval, a finding that may implicate X-chromosomal gene dosage effects on cardiac repolarisation rather than simple androgen deficiency (26). Collectively, these observations illustrate that cardiovascular risk in KS spans from congenital structural anomalies to complex arrhythmic susceptibility and premature mortality, underscoring the need for early and systematic cardiac evaluation in all patients with this syndrome.

### 3.3 Thromboembolism

One of the most clinically significant non-reproductive complications of KS is a profoundly elevated risk of venous thromboembolism (VTE). A Swedish nationwide cohort study following 1,085 men with KS between 1969 and 2010 calculated a standardised incidence ratio (SIR) for VTE of 6.43 (95% CI: 5.15-7.93), with the highest excess risk in younger age groups: SIR of 12.10 before age 30 years, and 11.00 between ages 30 and 49 years. The cumulative incidence of VTE was 8.6% by age 50 and 20.8% by age 70 (27).

In the large British cohort study of 3,518 KS patients, significantly elevated SMRs were recorded for pulmonary embolism (SMR 5.7), peripheral vascular disease (SMR 7.9), and cardiovascular disease overall (SMR 1.3). Mortality from ischaemic heart disease was, interestingly, lower than expected (SMR 0.7), possibly reflecting a protective hormonal effect or other unmeasured factors, the precise mechanism of which remains unclear (12).

The haemostatic abnormalities underlying this hypercoagulable state include reduced fibrin clot lysis, elevated fibrinogen and factor XIII levels, and increased plasminogen activator inhibitor type 1 (PAI-1) consistent with the hypofibrinolytic state attributable to androgen deficiency observed across the case series literature (27–29). Elevated factor VIII activity has also been reported in KS patients with venous ulceration, though this finding derives from case-level rather than systematic cohort data (30). The Factor VIII gene maps to chromosome Xq28, raising the possibility that gene dosage from the supernumerary X chromosome plays a direct, if as yet unproven, role in this prothrombotic phenotype. Pasquali et al. provided critical mechanistic insight by comparing KS men to men with hypogonadism from other causes receiving TRT: cardiovascular abnormalities resolved in the non-KS hypogonadal group but persisted in KS, suggesting that chromosomal architecture -rather than hypogonadism per se- underlies at least part of the cardiovascular risk in KS. This has important clinical implications, as it implies a non-modifiable chromosomal component that cannot be addressed by TRT alone (31). Collectively, these findings indicate that KS confers an intrinsic prothrombotic predisposition that is further amplified in the presence of additional genetic or acquired thrombophilic risk factors, and which warrants proactive clinical surveillance independent of hormonal status.

### 3.4 Management implications

All patients with KS should undergo cardiovascular risk assessment including blood pressure measurement, lipid profiling, and screening for congenital cardiac anomalies when clinically indicated. ECG monitoring for QTc abnormalities, particularly QTc shortening, which may predispose to sudden cardiac arrest, is appropriate, and at least one baseline 12-lead ECG is recommended per current guidelines. Clinicians should maintain a high index of suspicion for VTE, particularly in young men presenting with unexplained thrombosis, and consider thrombophilia workup and prophylaxis in high-risk situations. KS should be considered a genetic hypercoagulable state, analogous to inherited thrombophilias (16).

## 4 Bone health, sarcopenia, and the bone-testicular axis

### 4.1 Epidemiology of skeletal disease

Compromised bone health is a frequent and underappreciated complication of KS. Osteopenia occurs in 25-48% and frank osteoporosis in 6-15% of affected men. Fracture risk is elevated due to both reduced bone mineral density (BMD) and an increased predisposition to trauma. Bone turnover studies reveal a pattern of increased resorption with reduced formation, consistent with the hypogonadal state (32). Critically, BMD correlates poorly with serum testosterone levels in KS, challenging the assumption that hypogonadism is the sole driver of reduced bone mass (33, 34). Elevated FSH, which has been proposed to promote osteoclastogenesis via RANK upregulation and TNF-α stimulation, though clinical data in KS remain limited, unfavourable body composition, X chromosome inactivation patterns, and AR sensitivity all contribute to the skeletal fragility observed in KS (35–38).

This multifactorial pathophysiology is well reflected in the clinical literature. Van den Bergh et al. (39) provided early systematic data by measuring BMD and quantitative ultrasound parameters in 52 KS patients on long-term testosterone substitution, demonstrating that QUS indices at the calcaneus remained significantly lower than in age- and height-matched controls, establishing that skeletal compromise in KS persists even under androgen replacement therapy. Bojesen et al. (34) subsequently investigated a cohort of 70 adult KS patients and confirmed universally reduced BMD at the spine, hip and forearm compared to age-matched healthy subjects; multivariate analysis revealed that muscle strength, history of testosterone treatment, age at diagnosis, and bone turnover markers -rather than circulating testosterone levels per se- were the principal determinants of bone mass, while serum androgen concentration itself was not an independent predictor. Vogiatzi et al. (40) extended these observations to the paediatric setting in the first randomised, double-blind, placebo-controlled trial of oxandrolone in prepubertal boys with KS (ages 4–12 years, n=78), demonstrating a significant deficit in cortical bone mass at baseline and reporting that 13.5% of participants had a prior fracture history. Two years of oxandrolone treatment led to a significant improvement in bone mass relative to placebo, underscoring the importance of early androgen intervention during the critical window of skeletal development.

The molecular underpinnings of skeletal fragility in hypogonadal states have been illuminated by several mechanistic studies (41). Manolagas et al. (42) described how estrogen receptorα (Erα) and AR exert distinct and complementary effects on trabecular and cortical bone homeostasis. Estrogen-mediated protection of cortical bone mass operates via a non-nucleus-initiated ERα mechanism acting on osteoblasts, while ERα in osteoblast progenitors additionally stimulates Wnt signalling and periosteal bone accrual in response to mechanical strain, independently of estrogen. By contrast, AR signalling in mature osteoblasts is indispensable for the maintenance of trabecular bone mass in male mammals, though it is not required for the anabolic effects of androgens on cortical bone. Complementing these findings, Chen et al. (43), in a comprehensive synthesis of *in vitro* and *in vivo* evidence, demonstrated that androgens promote osteoblastic mineralisation through AR-mediated upregulation of tissue non-specific alkaline phosphatase and SIBLING family proteins, thereby ensuring sufficient phosphate availability for hydroxyapatite crystal formation; androgen deficiency, as seen in male hypogonadism, is accordingly associated with impaired osteoblastic mineralisation and accelerated trabecular bone resorption, translating into clinically significant bone loss and increased fracture risk.

### 4.2 The INSL3-RXFP2-osteocalcin axis

Emerging research has implicated insulin-like peptide 3 (INSL3), a Leydig cell-specific hormone, as a key regulator of bone metabolism in KS. INSL3 acts on osteoblasts through its receptor RXFP2, activating adenylate cyclase and increasing intracellular cAMP, as well as engaging the MAPK signalling cascade. This leads to osteoblast proliferation, differentiation and maturation including upregulation of alkaline phosphatase and other mineralisation-related markers and ultimately to mineralisation of the extracellular matrix. Additionally, INSL3 acts directly on osteocytes via RXFP2 to suppress sclerostin (SOST) expression; since sclerostin is a potent Wnt antagonist, its downregulation disinhibits Wnt-driven bone formation and indirectly attenuates osteoclastogenesis (44–46). In men with KS, INSL3 levels are markedly reduced compared to age-matched controls, with testosterone replacement therapy further suppressing circulating concentrations. Studies in KS cohorts have demonstrated that INSL3 levels positively correlate with total osteocalcin and negatively with sclerostin. The relationship between INSL3 and BMD remains inconsistent across studies, suggesting that INSL3 may influence bone quality through mechanisms not fully captured by conventional densitometric assessment (47).

The concept of the bone-testicular axis has added another layer of complexity to the skeletal pathophysiology of KS. Osteocalcin which is a bone matrix protein that also functions as an endocrine hormone stimulates testicular steroidogenesis by binding to the GPRC6A receptor on Leydig cells, thereby upregulating steroidogenic gene expression and modulating LH receptor signalling. In a large retrospective longitudinal KS cohort (n=254), total osteocalcin was highest in eugonadal men and significantly lower in TRT recipients, and was directly associated with both LH (p=0.017) and FSH (p=0.004) levels in adult patients. TRT suppressed osteocalcin concentrations significantly within 3 months of initiation (p=0.006), suggesting that exogenous testosterone exerts a negative feedback effect on this bone-testicular axis (48).

### 4.3 FSH and bone resorption

Elevated FSH levels, a constant feature of KS, independently drive bone resorption. Chin et al. (35), in a comprehensive review published in the International Journal of Medical Sciences, confirmed that FSH directly stimulates osteoclast formation, function and survival via a Gi-coupled FSH receptor expressed on osteoclast precursors, increases RANK expression and TNF-α production within the bone marrow microenvironment, promotes osteoclast differentiation and resorptive activity, and prevents osteoclast apoptosis, providing a mechanism by which the chronically elevated FSH in KS may contribute to bone loss independently of sex steroid levels. The transdermal route of TRT administration, unlike long-acting testosterone undecanoate injections, does not suppress FSH, with LH and FSH levels remaining significantly higher in the transdermal group (p=0.002), which has theoretical implications for long-term BMD outcomes; however, a 5-year retrospective study of 147 KS males comparing TRT routes found no significant differences in hip or spine BMD between groups (49).

### 4.4 The TRAVERSE trial and fracture risk

The fracture subtrial of the double-blind, randomised, placebo-controlled TRAVERSE trial evaluated fracture outcomes in 5,204 hypogonadal men (aged 45–80 years) over a median follow-up of 3.19 years. Clinical fractures occurred in 3.50% of the testosterone group versus 2.46% of the placebo group (HR 1.43, 95% CI 1.04-1.97), with the cumulative incidence rising to 3.8% versus 2.8% by year 3. Notably, over 80% of fractures occurred in the context of trauma, most commonly falls, and the most frequent sites were the ribs, wrist and ankle. Although these results were not generated specifically in KS patients and cannot be directly extrapolated given the differing pathophysiology they nonetheless underscore the need for careful fracture risk monitoring during TRT, particularly in those with pre-existing bone disease (50).

### 4.5 Management recommendations

EAA 2021 guidelines recommend DXA scanning at the lumbar spine and femoral neck for all adult patients with KS at the first visit, with fracture risk assessment and subsequent monitoring on an individual basis (16). Recent literature additionally suggests that DXA alone may not fully characterise bone quality in KS, and that vertebral fracture assessment independent of BMD results and trabecular bone score should be considered as complementary tools (51). Vitamin D supplementation is indicated given the high prevalence of 25-OHD deficiency in this population, with evidence that vitamin D repletion may be more effective than TRT alone in restoring BMD (52). Sarcopenia assessment using validated tools including the SARC-F questionnaire, grip strength, the chair stand test, and appendicular skeletal muscle mass by DXA and resistance exercise prescription are encouraged (52). Anti-resorptive therapy should be guided by testosterone status: bisphosphonates represent the primary pharmacological option in eugonadal KS patients with low BMD, while in hypogonadal patients with high fracture risk, anti-resorptive agents should be combined with TRT when testosterone replacement alone is insufficient to restore bone density (16).

## 5 Thyroid dysfunction

Thyroid dysfunction in KS remains insufficiently characterised but appears to involve subtle yet clinically meaningful alterations in hypothalamic-pituitary-thyroid axis regulation. A Danish case-control study comparing 75 men with KS to 75 age-matched healthy controls demonstrated significantly reduced fT4 concentrations with a correspondingly lower freeT4/freeT3 ratio and no compensatory rise in TSH, leading the authors to conclude that an inadequate hypothalamic-pituitary control of thyroid function is a feature of KS, most likely reflecting an altered T4:TSH set point rather than primary thyroid failure (53). Carlomagno et al. (54), in a comprehensive retrospective longitudinal study of 254 KS patients spanning infancy to adulthood, confirmed that free thyroid hormones are reduced across all pubertal stages and TSH values being lower only in adults, and that peripheral sensitivity to thyroid hormones remains intact pointing instead to a central feedback dysregulation. Crucially, *in vitro* testing in this study demonstrated an inhibitory effect of testosterone on pituitary type 2 deiodinase expression and activity, supporting the hypothesis that in male hypogonadism the pituitary sensing of circulating thyroid hormones is enhanced thereby explaining the inappropriately low TSH relative to circulating thyroid hormone levels in KS.

An increased prevalence of autoimmune thyroid disease has also been described in KS, consistent with the broader immune dysregulation associated with sex chromosome aneuploidy. Complementing these functional findings, di Fraia et al. (55) reported a significantly higher prevalence of nodular thyroid disease in 122 KS patients compared to 85 age-matched controls (31% vs 13%), with all biopsied nodules confirmed cytologically benign. The authors proposed that chronically low fT4 levels, inappropriate TSH secretion, and genetic instability related to the supernumerary X chromosome may collectively drive this structural thyroid pathology, findings that further support the concept of KS as a systemic disorder extending beyond endocrine dysfunction. Given the overlap between thyroid dysfunction and several KS-related comorbidities including metabolic and neurocognitive disturbances routine thyroid screening may be clinically justified, although evidence-based management strategies remain limited.

## 6 Neurocognitive and psychiatric features

Neurocognitive and psychological features represent a core component of the KS phenotype. Structural and functional neuroimaging studies have consistently demonstrated alterations in brain regions involved in language processing, executive function, and social cognition. Clinically, individuals with KS frequently exhibit impairments in verbal abilities, language development, and reading skills, while non-verbal intelligence is relatively preserved. Increased rates of attention deficit/hyperactivity disorder, anxiety, depression, and autism spectrum traits have also been reported. These features are likely driven by both androgen deficiency and direct gene-dosage effects influencing neurodevelopment (56).

The neurocognitive profile of KS is broad and heterogeneous, extending well beyond language difficulties alone. Deficits are present across multiple cognitive domains of varying severity, with general cognitive abilities, language, and executive functioning characteristically affected, while social dysfunction is also frequently observed. Notably, dyslexia has been reported in more than half of all affected males. Neurological comorbidities further compound this burden: neurological disorders among males with KS are significantly elevated, with epilepsy carrying a hazard ratio of approximately 4.28 and cerebral palsy or paresis a hazard ratio of around 2.89 compared with the general population (56). Global epigenetic and RNA expression changes are now understood to play a central role in shaping the KS phenotype (56), suggesting that the neurocognitive manifestations of the syndrome arise from deeply rooted genomic dysregulation rather than hormonal deficiency alone (56).

The neurodevelopmental burden is further amplified in higher-grade X chromosome aneuploidies. Tartaglia et al. (57) demonstrated that 48,XXYY, 48,XXXY and 49,XXXXY syndromes should not be regarded merely as variants of KS, but as distinct clinical entities with progressively more severe neurocognitive, behavioural, and developmental profiles underscoring the principle that each additional X chromosome confers an incremental burden on neurodevelopment. In this context, Samango-Sprouse et al. provided evidence that early androgen intervention may be beneficial across the spectrum of X chromosome aneuploidies: a short course of androgen therapy in infants with 49,XXXXY syndrome resulted in measurable improvements in speech and language, gestural communication and vocabulary development (58); and separately, early androgen treatment in boys with 47,XXY at 36 and 72 months of age yielded significant improvements in neurodevelopmental outcomes including language acquisition and motor development (59) together suggesting that the neurodevelopmental trajectory in X chromosome aneuploidies may be modifiable if treatment is initiated early.

Increasing evidence suggests that many non-reproductive features of KS originate during childhood, well before the onset of overt hypogonadism. Early metabolic alterations, including increased truncal adiposity and elevated rates of metabolic syndrome, neurodevelopmental differences, and subtle endocrine abnormalities may already be present in prepubertal individuals, highlighting the importance of early diagnosis and longitudinal follow-up (3, 7). Early identification and targeted interventions including speech therapy, educational support, and psychological care are essential to optimise long-term outcomes. Clinicians should screen systematically for neurocognitive and psychiatric comorbidities as part of a comprehensive, multidisciplinary management strategy (16).

From a clinical management perspective, a multidisciplinary approach to KS treatment spanning from childhood through to senescence is strongly advocated, and neonatal screening programmes have been proposed as a means of reducing the currently unacceptably high rates of missed or delayed diagnosis, with an estimated 50-75% of affected males never receiving a formal diagnosis (3, 16). Future studies will need to evaluate the effects of TRT on both metabolic risk and neurocognitive outcomes (3), as the evidence base in this area remains limited. The neurocognitive phenotype of KS is increasingly being characterised, and the need for psychological and cognitive interventions in many cases is now well-recognised (56); yet translation of this knowledge into systematic, guideline-driven clinical practice remains inconsistent across centres. Taken together, these considerations reinforce the imperative for early, proactive, and coordinated care that addresses not only the endocrine but also the neuropsychiatric dimensions of the syndrome.

## 7 Malignancy risk

KS is associated with a distinct pattern of altered malignancy risk. Male breast cancer is markedly elevated, with standardised incidence ratios ranging from 14 to 50 in different cohort studies, reflecting the combination of relative hyperoestrogenaemia, gynaecomastia, and potentially direct X-chromosomal gene-dosage effects on mammary tissue (60, 61). This risk warrants routine breast examination and a low threshold for further investigation of breast symptoms.

Haematological malignancies represent an area of incompletely resolved risk. Non-Hodgkin lymphoma is reported at increased frequency in KS, with elevated mortality demonstrated in large cohort studies, particularly among men with a 48,XXYY constitution (60). Leukaemia has been described in case series and case reports; however, it has not been consistently confirmed as a statistically significant excess risk in cohort-level data, and its association with KS should therefore be interpreted with caution (60). Extragonadal germ cell tumours, particularly mediastinal nonseminomatous germ cell tumours, occur at substantially higher rates than in the general male population. Their pathogenesis in KS is considered multifactorial: residual primordial germ cells retained along the urogenital ridge during embryogenesis provide the cellular substrate, while the hypergonadotropic state characteristic of KS is hypothesised to exert chronic stimulatory effects on these extragonadal germ cells (60, 62).

Conversely, prostate cancer risk appears to be significantly reduced in KS, likely reflecting the sustained androgen-deficient milieu combined with elevated oestrogen levels, which exert protective effects on prostatic epithelium. This observation has generated interest in the hormonal regulation of prostate carcinogenesis, though it does not modify recommendations regarding PSA monitoring during TRT (60).

Overall, clinicians should apply cancer surveillance strategies adapted to this altered risk profile, ensuring breast examination and haematological monitoring are incorporated into routine follow-up, while maintaining standard age-appropriate cancer screening for other malignancies.

## 8 Autoimmunity, accelerated biological ageing, and mortality

Individuals with KS demonstrate a broad susceptibility to autoimmune disorders that extends well beyond the thyroid axis. Increased prevalences have been documented for systemic lupus erythematosus (SLE), T1DM, rheumatoid arthritis, and Sjögren syndrome. The excess risk of SLE in men with KS is particularly striking, as this condition is otherwise predominantly female, and its occurrence in 47,XXY individuals directly implicates X-chromosomal gene-dosage in immune dysregulation (14, 15, 62, 63).

The mechanisms underlying immune dysregulation in KS include overexpression of X-linked immune regulatory genes that escape inactivation, altered sex hormone profiles -oestrogen exerting pro-inflammatory effects in certain immune pathways- and disruption of central and peripheral tolerance mechanisms. These findings position KS as a model for understanding sex chromosome contributions to autoimmunity more broadly (62, 63).

Accumulating evidence indicates that KS is associated with an altered trajectory of biological ageing at the cellular level. Men with KS exhibit longer telomeres than age-matched controls in early adulthood; however, the rate of telomere attrition with advancing age is substantially steeper in KS than in both XY males and XX females, suggesting accelerated replicative senescence in later decades. Whether DNA methylation-based epigenetic clock analyses consistently demonstrate accelerated biological ageing in KS remains an area of active investigation, with current data yielding heterogeneous results across different clock algorithms (64).

These mechanisms are reflected in population-level mortality data. The landmark British cohort study of 3,518 KS patients reported an all-cause SMR of 1.5 (95% CI 1.4-1.7), corresponding to an estimated median reduction in life expectancy of approximately 2 years. Cause-specific excess mortality was driven by respiratory disease (SMR 2.3), diabetes (SMR 5.8), pulmonary embolism (SMR 5.7), and peripheral vascular disease (SMR 7.9). The relative underrepresentation of ischaemic heart disease mortality remains an intriguing finding, potentially attributable to the cardioprotective effects of elevated oestrogen levels (12).

## 9 Karyotypic heterogeneity and phenotypic variability

An important yet underemphasised aspect of KS is the heterogeneity of clinical presentation according to karyotype. The classic 47,XXY constitution accounts for approximately 90% of cases, but this figure conceals considerable biological and phenotypic diversity. Mosaic forms (46,XY/47,XXY) typically display milder phenotypes: testosterone levels may be higher, spermatogenesis better preserved, and the burden of metabolic and neurocognitive comorbidities substantially lower. This variability underscores the importance of karyotype documentation at diagnosis, as management intensity and surveillance frequency may need to be calibrated accordingly (3, 4).

At the other extreme, higher-grade aneuploidies, 48,XXXY and 49,XXXXY, are associated with progressively more severe phenotypes, including profound intellectual disability, marked dysmorphic features, severe hypogonadism, and multi-organ involvement. These conditions are sufficiently distinct in their neurological and developmental burden to warrant separate clinical consideration, and their management requires subspecialty expertise in genetics, developmental paediatrics, and rehabilitation medicine (57).

The mechanisms underlying this phenotypic gradient are not simply linear with X chromosome copy number. Gene-dosage effects interact with epigenetic regulation, particularly X-inactivation patterns and DNA methylation, in ways that remain incompletely characterised. The proportion of genes escaping X-inactivation may itself vary between individuals with the same karyotype, providing one plausible explanation for inter-individual variability within classical 47,XXY KS. Future research employing transcriptomic and epigenomic profiling across karyotype groups will be essential to delineate these mechanisms (5, 6).

## 10 Sexual health and non-reproductive andrological features

Although infertility and testicular dysfunction are the most widely recognised andrological features of KS, the non-reproductive aspects of sexual health are frequently neglected in clinical practice. Erectile dysfunction (ED) is reported at increased frequency in men with KS and is multifactorial in origin. Importantly, ED in KS does not appear to be primarily driven by androgen deficiency per se; penile vascular function is typically preserved on Doppler assessment, and the oestradiol-to-testosterone ratio rather than testosterone alone has been identified as the principal hormonal predictor of erectile function. Psychological disturbance, present at high prevalence in KS men with ED, appear to be a major contributing factor (65, 66).

Reduced libido is the most consistently reported sexual complaint across KS cohorts, affecting approximately 55% of men with KS compared with around 17% of age-matched controls, and is more directly associated with testosterone levels than ED. Notably, TRT improves sexual desire, intercourse satisfaction, and overall satisfaction, but does not restore erectile function, underscoring the distinct pathophysiological mechanisms underlying these two aspects of sexual dysfunction (65, 66). Gynaecomastia, resulting from the elevated oestrogen-to-androgen ratio characteristic of KS, is present in approximately 70-80% of affected individuals. Beyond its cosmetic impact, gynaecomastia is associated with significant psychosocial distress and contributes to the elevated breast cancer risk observed in KS. Surgical management (subcutaneous mastectomy) may be appropriate in symptomatic or persistent cases and should be considered as part of the multidisciplinary care plan (67).

Penile size and testicular volume are typically reduced in KS, and these physical characteristics may be sources of significant personal distress, particularly during adolescence. Clinicians should proactively address sexual health concerns in a sensitive and non-judgmental manner, and access to specialist psychosexual counselling should be facilitated when indicated (68).

## 11 Testosterone replacement therapy: systemic considerations

TRT remains the cornerstone of long-term management for hypogonadism in KS and should be initiated at puberty or upon diagnosis in adulthood. The primary goals of TRT extend beyond sexual function: treatment aims to restore eugonadal testosterone levels in order to preserve bone mineral density, optimise body composition, support mood and cognitive function, and reduce the metabolic burden of androgen deficiency. Evidence from systematic review and meta-analysis confirms that TRT improves body composition and spinal bone mineral density in KS; however, it does not normalise lipid profile or glycaemic control, and treated KS patients continue to demonstrate worse metabolic parameters than age-matched eugonadal controls. This persistent residual risk underscores the principle that TRT addresses the hormonal but not the chromosomal substrate of systemic disease in KS (16, 69).

The choice of TRT formulation warrants individualised consideration. Long-acting intramuscular testosterone undecanoate and transdermal gel preparations are the most widely used modalities in clinical practice. Real-world data from a five-year observational cohort demonstrate that both routes of administration achieve comparable testosterone levels and produce similar effects on haematocrit, bone mineral density, lipid profile, and glycaemic parameters; neither formulation has been shown to confer clear metabolic superiority. Clinician and patient preference, adherence, and individual risk factors including VTE risk and haematocrit trajectory should therefore guide formulation selection (16, 49). Notably, TRT does not appear to independently increase VTE risk in KS when testosterone levels are maintained within the physiological range, though the underlying hypercoagulable state demands ongoing vigilance. TRT may in fact confer indirect haemostatic benefit through reduction of fat mass, which is itself a predictor of VTE (70).

Monitoring during TRT should be systematic and structured. At minimum, surveillance should encompass serum testosterone (targeting mid-normal range), haematocrit, PSA, fasting lipids, fasting glucose or HbA1c, and bone mineral density at appropriate intervals. Blood pressure monitoring and assessment of thrombotic risk markers should be incorporated given the elevated cardiovascular and thromboembolic morbidity profile of KS. Given that chromosomal mechanisms drive a substantial proportion of systemic risk independently of testosterone levels, TRT should be viewed as necessary but not sufficient, a platform upon which additional targeted surveillance and risk-reduction interventions must be built (16).

Advances in reproductive techniques have fundamentally altered the counselling landscape for men with KS. Testicular sperm extraction (TESE), particularly microdissection TESE, combined with intracytoplasmic sperm injection (ICSI) achieves sperm retrieval rates of approximately 50% in non-mosaic 47,XXY men, with subsequent live birth rates of similar magnitude. These outcomes are largely independent of pretreatment FSH, LH, or testosterone levels, making it difficult to identify reliable predictors of success. The possibility of biological paternity carries significant psychosocial implications and should be introduced into management discussions from adolescence onwards, including counselling regarding fertility preservation options prior to TRT initiation where appropriate (16, 71).

## 12 Non-pharmacological interventions and lifestyle management

Pharmacological management, centred on TRT and metabolic risk factor treatment, represents only one dimension of comprehensive KS care. Non-pharmacological interventions addressing physical, cognitive, and psychosocial domains are equally essential yet chronically underutilised in clinical practice, and their integration into care pathways from the time of diagnosis should be considered a clinical standard rather than an adjunct.

Structured physical activity particularly resistance exercise training has a well-established evidence base in hypogonadal men for improving lean body mass, insulin sensitivity, and bone mineral density. In KS specifically, muscle strength has been identified as an independent predictor of bone mineral density, separate from testosterone levels, underscoring the role of exercise beyond its indirect androgenic effects. Weight-bearing and resistance exercise should therefore be actively prescribed as part of metabolic risk management, and referral to exercise physiology services should be considered at diagnosis and at follow-up intervals (16, 72).

Dietary intervention is a cornerstone of metabolic risk reduction in KS. Visceral adiposity, prevalent in untreated and even treated KS patients, drives insulin resistance, dyslipidaemia, and cardiovascular risk independently of testosterone status. Energy restriction strategies targeting abdominal obesity, alongside dietary patterns characterised by high intake of unsaturated fats, fibre, and antioxidants, are particularly appropriate given the pro-inflammatory metabolic phenotype of KS. Dietetic referral should be offered as standard at diagnosis and revisited longitudinally, ideally within a structured multidisciplinary framework (8, 11).

Cognitive and neuropsychological support represents a clinically important but systematically underserved domain. Approximately 80% of boys with KS require some form of specialised educational support for language-based learning difficulties, and speech and language therapy is indicated for all children and adolescents demonstrating speech, language, literacy, or social-pragmatic deficits. Early detection of phonological errors and prompt initiation of speech-language therapy are associated with better academic and social outcomes, and speech-language evaluation should be embedded in the routine paediatric care pathway for all children with KS (72, 73). Beyond language, structured neuropsychological programmes targeting executive function, working memory, and processing speed merit formal evaluation in KS; evidence from adjacent neurodevelopmental populations supports their potential utility, though KS-specific trial data remain limited.

Psychological and psychotherapeutic support should be routinely offered at all stages of the lifespan rather than reserved for cases of severe psychiatric comorbidity. Cognitive behavioural therapy is an evidence-based modality for anxiety and depression conditions that are prevalent in KS and psychosexual counselling addresses the sexual health concerns that are frequently present but rarely proactively discussed. Peer support through patient advocacy organisations provides an additional layer of psychosocial benefit, mitigating the social isolation that commonly follows diagnosis. Quality of life data consistently demonstrate that psychological distress, personality traits, and inadequate coping strategies contribute substantially to reduced wellbeing in KS beyond what is explained by hormonal deficiency alone, reinforcing the need for psychological intervention as a core rather than supplementary component of care (68, 74).

Finally, health behaviour counselling should be proactively integrated into KS management. Smoking cessation is particularly relevant given the already-elevated cardiovascular and thromboembolic risk profile of KS, and pharmacological cessation support should be offered where appropriate. Alcohol moderation should be discussed in the context of its effects on testosterone metabolism and liver function. In older men with KS, the convergence of sarcopenia, reduced proprioception, and elevated fracture risk warrants targeted fall prevention strategies and balance training as part of a holistic musculoskeletal care plan (16).

## 13 The paediatric window: early diagnosis and intervention

A recurring theme across the KS literature is that many of the condition’s most significant non-reproductive manifestations originate well before the onset of clinically overt hypogonadism. Prepubertal boys with KS demonstrate subtle but measurable differences in body composition, insulin sensitivity, neurodevelopmental trajectory, and gonadal function compared to age-matched controls. This observation carries profound implications: it means that by the time classical features of hypogonadism emerge in adolescence or adulthood, a window of preventive opportunity may already have partly closed (7, 75).

The case for early diagnosis is therefore not limited to the reproductive domain. Identification of KS in infancy or early childhood, whether through prenatal diagnosis, neonatal screening, or recognition of early developmental features, enables timely neurodevelopmental intervention, metabolic surveillance, and psychological support during critical developmental periods. Language delay and learning difficulties, if identified and addressed early, have a substantially better prognosis than when intervention is delayed until school age or beyond (76, 77).

Early androgen supplementation in infancy (mini-puberty augmentation) or childhood has been explored as a means of promoting neurodevelopmental outcomes in some centres, though evidence for this approach remains limited and its long-term effects on reproductive potential and bone metabolism require careful evaluation. This strategy should currently be considered investigational and undertaken only within research frameworks with robust follow-up (75).

The diagnostic pathway in paediatric KS deserves specific attention. Many clinicians outside specialist centres remain unfamiliar with the non-reproductive features of KS in children, and referral patterns often focus narrowly on growth concerns or genital examination findings. Increasing awareness among general paediatricians, child psychologists, and educational professionals is essential to shorten the diagnostic delay that characterises KS across all age groups (4, 77).

## 14 Psychosocial impact and quality of life

The psychosocial burden of KS is substantial and encompasses dimensions that extend well beyond the direct clinical manifestations of the condition. The experience of receiving a diagnosis which is often delayed until adulthood and frequently precipitated by investigation for infertility is in itself psychologically significant. Many men report feelings of shock, grief, and altered identity upon diagnosis, and the associated infertility may have profound effects on relationships and sense of self (68).

Stigma, both real and perceived, represents an important barrier to help-seeking and treatment engagement. KS remains poorly understood outside specialist clinical settings, and affected individuals frequently report experiences of dismissal or minimisation by healthcare providers, as well as social isolation arising from difficulties in disclosing their condition. The relatively low public profile of KS, compared with other genetic conditions of similar prevalence, compounds this experience (68).

Health-related quality of life (HRQoL) is consistently reduced in men with KS across multiple domains, including physical functioning, vitality, sexual satisfaction, and mental health, as measured by validated instruments such as the SF-36 and WHOQOL-BREF. Importantly, HRQoL does not uniformly improve with TRT alone, reinforcing the need for psychological support as a core component of management rather than an adjunct (68, 78).

Fatigue is one of the most prevalent and impactful symptoms reported by men with KS, yet it is frequently under-addressed in clinical encounters. Its aetiology is likely multifactorial, encompassing hypogonadism, sleep disturbance (including increased rates of obstructive sleep apnoea), depression, and anaemia. Systematic enquiry about fatigue and its functional consequences should be incorporated into routine clinical assessment (78, 79).

Chronic musculoskeletal pain, particularly in the lower limbs, is reported more frequently in KS than in age-matched controls and may relate to a combination of sarcopenia, skeletal fragility, and altered gait mechanics. This symptom domain has received little research attention and represents an important unmet clinical need. Patient-reported outcome measures should be incorporated into clinical research and practice to ensure that the full experience of living with KS is captured and addressed (79).

## 15 Multidisciplinary surveillance framework

Given the broad systemic involvement of KS, a structured, multidisciplinary surveillance strategy is essential. The following framework reflects current EAA 2021 guidelines and the expanded evidence base discussed in this review (16).

Metabolic and cardiovascular surveillance should include annual assessment of body composition, fasting plasma glucose, HbA1c, lipid profile, and blood pressure from early adulthood. Cardiovascular risk assessment should include ECG monitoring and, where clinically indicated, echocardiography to screen for congenital cardiac anomalies. VTE risk should be formally assessed and documented, particularly before surgery, immobilisation, or hormonal changes.

Skeletal health monitoring should include DXA scanning with trabecular bone score assessment at diagnosis and at regular intervals. Vitamin D status should be evaluated and supplemented. Sarcopenia should be assessed using validated tools, and fall risk evaluated in older patients.

Endocrine monitoring should encompass thyroid function testing (including TPO and anti-thyroglobulin antibodies), regular assessment of testosterone and gonadotrophin levels, and monitoring of TRT adequacy and safety parameters including haematocrit and PSA.

Sexual and andrological assessment should be performed routinely, including systematic enquiry about erectile function, libido, and sexual satisfaction. Gynaecomastia should be documented and, where symptomatic or psychologically distressing, surgical referral offered. Psychosexual counselling should be available.

Neurocognitive and psychological evaluation should be performed at diagnosis and in childhood when KS is identified early. Access to speech therapy, educational support, and psychological or psychiatric care should be facilitated. Fatigue and chronic pain should be specifically enquired about and addressed.

Oncological surveillance should be adapted to the altered malignancy risk profile of KS, with routine breast examination and haematological monitoring incorporated into follow-up. Standard age-appropriate cancer screening recommendations should continue to apply.

Psychosocial support should be offered proactively, including signposting to patient support organisations, peer networks, and, where appropriate, psychotherapy. HRQoL should be formally assessed at intervals using validated instruments.

## 16 Emerging concepts and future perspectives

Despite significant advances in understanding the multisystem burden of KS, several critical knowledge gaps persist. The relative contribution of genetic versus hormonal mechanisms across different organ systems remains incompletely resolved, limiting the development of targeted non-hormonal therapies. The long-term impact of early testosterone therapy on neurodevelopment, bone metabolism, and cardiovascular risk requires evaluation in prospective paediatric cohorts (72, 75).

Biomarker research represents a priority area. INSL3, as a Leydig cell-specific hormone that reflects gonadal functional reserve independently of testosterone, holds promise as a sensitive indicator of gonadal status and bone metabolic function. Epigenetic biomarkers including DNA methylation-based biological age estimators may prove useful for identifying individuals at highest risk of accelerated comorbidity onset and for monitoring therapeutic response (44, 47, 64).

The field of non-pharmacological intervention in KS is substantially underdeveloped. Randomised controlled trials evaluating structured exercise programmes, dietary interventions, and cognitive rehabilitation in well-characterised KS cohorts are urgently needed. Similarly, psychosocial interventions including peer support, psychotherapy, and patient education programmes require rigorous evaluation using validated HRQoL endpoints (68, 72).

Prospective, longitudinal cohort studies specifically designed for KS patients rather than extrapolated from general hypogonadal male populations are essential to define optimal surveillance intervals, identify predictors of adverse outcomes, and evaluate the long-term effectiveness of current management strategies. International collaboration and data harmonisation will be required to generate cohorts of sufficient size to address these questions (16).

Finally, the healthcare system and societal dimensions of KS require attention. Reducing the diagnostic delay, currently averaging more than two decades from birth, demands educational initiatives targeting clinicians across multiple specialties, including general practice, paediatrics, urology, and psychiatry. Integrating KS into medical curricula and continuing professional development programmes represents a readily actionable step toward improving population-level outcomes for this underdiagnosed condition (2, 4).

## 17 Conclusion

KS should be recognised as a complex multisystem disorder rather than a condition confined to reproductive dysfunction. The presence of an additional X chromosome results in a broad spectrum of metabolic, cardiovascular, skeletal, endocrine, andrological, oncological, autoimmune, and neurocognitive abnormalities, mediated through both hormonal perturbation and direct chromosomal gene-dosage effects.

While TRT remains essential and addresses certain aspects of hypogonadism, it neither fully mitigates the systemic burden associated with KS nor substitutes for comprehensive surveillance and multidisciplinary care. Emerging insights into novel pathways the INSL3-mediated bone axis, osteocalcin-driven endocrine crosstalk, accelerated epigenetic ageing, and X-chromosomal immune dysregulation highlight an expanding therapeutic landscape that extends well beyond androgen replacement.

Karyotypic heterogeneity must be acknowledged in clinical practice, with management intensity calibrated to individual phenotypic burden. Non-pharmacological interventions like structured exercise, dietary optimisation, cognitive rehabilitation, and psychosocial support deserve equal prominence alongside pharmacotherapy in the management framework. The paediatric window of opportunity for early intervention must not be overlooked, and the psychosocial dimensions of KS must be systematically and sensitively addressed.

From a public health perspective, increasing awareness among clinicians across all specialties remains the most critical and actionable priority. Given that up to 75% of individuals with KS remain undiagnosed throughout their lives, earlier recognition and earlier implementation of the surveillance and intervention strategies described in this review represents the most immediate pathway to improving long-term health outcomes and quality of life in this population.

In addition to endocrine abnormalities, individuals with Klinefelter syndrome exhibit an increased risk of thromboembolic and cardiovascular complications, potentially mediated by hormonal imbalance, altered coagulation pathways, and metabolic disturbances. Neurocognitive and psychiatric features, including language impairment, executive dysfunction, and increased susceptibility to mood disorders, are also observed.

Furthermore, gene dosage effects involving X-linked immune-related genes may contribute to immune dysregulation, leading to increased susceptibility to autoimmune diseases. Testosterone replacement therapy (TRT) may partially ameliorate some of these features, particularly metabolic and bone-related outcomes; however, its effects on cardiovascular and thrombotic risk remain complex and require individualized assessment.

Overall, Klinefelter syndrome should be recognized as a complex multisystem disorder rather than a condition limited to hypogonadism.
